# ﻿Karyotype differentiation in the *Nothobranchiusugandensis* species group (Teleostei, Cyprinodontiformes), seasonal fishes from the east African inland plateau, in the context of phylogeny and biogeography

**DOI:** 10.3897/compcytogen.v7.i1.97165

**Published:** 2023-01-31

**Authors:** Eugene Yu. Krysanov, Béla Nagy, Brian R. Watters, Alexandr Sember, Sergey A. Simanovsky

**Affiliations:** 1 Severtsov Institute of Ecology and Evolution, Russian Academy of Sciences, Leninsky Prospect 33, 119071, Moscow, Russia Severtsov Institute of Ecology and Evolution, Russian Academy of Sciences Moscow Russia; 2 15, voie de la Liberté, 77870, Vulaines sur Seine, France Unaffiliated Vulaines sur Seine France; 3 6141 Parkwood Drive, Nanaimo, British Columbia V9T 6A2, Nanaimo, Canada Unaffiliated Nanaimo Canada; 4 Laboratory of Fish Genetics, Institute of Animal Physiology and Genetics, Czech Academy of Sciences, Rumburská 89, 27721, Liběchov, Czech Republic Laboratory of Fish Genetics, Institute of Animal Physiology and Genetics, Czech Academy of Sciences Liběchov Czech Republic

**Keywords:** 2n uniformity, chromosomes, chromosome evolution, chromosome inversion, cytogenetics, karyotype variability

## Abstract

The karyotype differentiation of the twelve known members of the *Nothobranchiusugandensis* Wildekamp, 1994 species group is reviewed and the karyotype composition of seven of its species is described herein for the first time using a conventional cytogenetic protocol. Changes in the architecture of eukaryotic genomes often have a major impact on processes underlying reproductive isolation, adaptation and diversification. African annual killifishes of the genus *Nothobranchius* Peters, 1868 (Teleostei: Nothobranchiidae), which are adapted to an extreme environment of ephemeral wetland pools in African savannahs, feature extensive karyotype evolution in small, isolated populations and thus are suitable models for studying the interplay between karyotype change and species evolution. The present investigation reveals a highly conserved diploid chromosome number (2n = 36) but a variable number of chromosomal arms (46–64) among members of the *N.ugandensis* species group, implying a significant role of pericentric inversions and/or other types of centromeric shift in the karyotype evolution of the group. When superimposed onto a phylogenetic tree based on molecular analyses of two mitochondrial genes the cytogenetic characteristics did not show any correlation with the phylogenetic relationships within the lineage. While karyotypes of many other *Nothobranchius* spp. studied to date diversified mainly via chromosome fusions and fissions, the *N.ugandensis* species group maintains stable 2n and the karyotype differentiation seems to be constrained to intrachromosomal rearrangements. Possible reasons for this difference in the trajectory of karyotype differentiation are discussed. While genetic drift seems to be a major factor in the fixation of chromosome rearrangements in *Nothobranchius*, future studies are needed to assess the impact of predicted multiple inversions on the genome evolution and species diversification within the *N.ugandensis* species group.

## ﻿Introduction

The cyprinodontiform fish genus *Nothobranchius* Peters, 1868 currently comprises 96 valid species, occurring mainly in seasonal wetlands of river drainages in north-eastern, eastern and south-eastern Africa that are subject to seasonal rainfall (Nagy and Watters 2021). All known species feature an annual or semi-annual life cycle as a key adaptation to reproduce in an unpredictable biome of temporary freshwater pools that appear during monsoons, and which become desiccated during the dry season (Vanderplank 1940; Watters 2009; Nagy 2015). Because of their life cycle, annual killifishes form small populations with non-overlapping generations that are biogeographically isolated. Their low dispersal ability leads to strong spatial genetic structure of *Nothobranchius* spp. (Bartáková et al. 2013, 2015; Dorn et al. 2014) with a strong effect of genetic drift, including bottlenecks and founder effects, on their genome evolution (Bartáková et al. 2013; Cui et al. 2019; van der Merwe et al. 2021).

**Figure 1.**
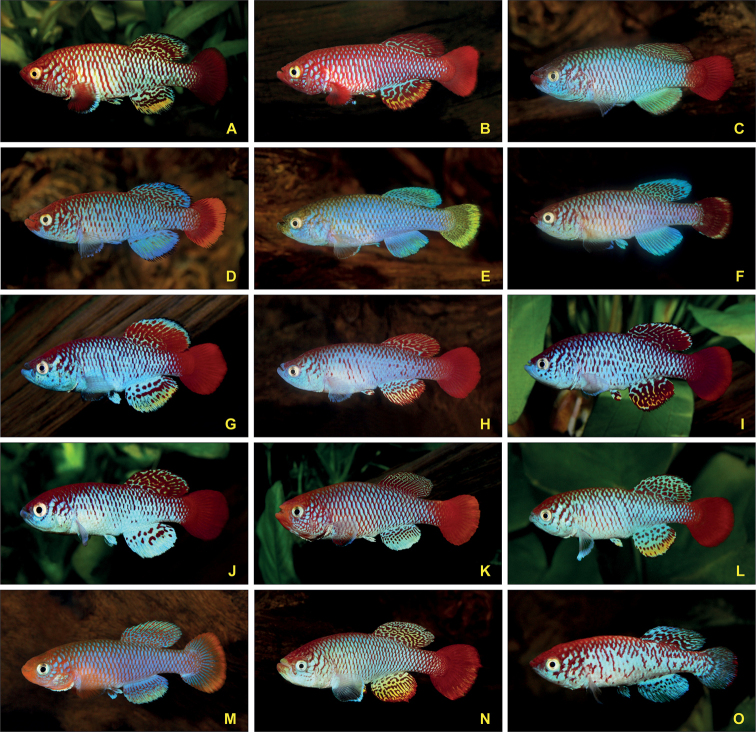
Selected male specimens of representatives of the *Nothobranchiusugandensis* species group (*denotes populations from which karyotype data was determined) **A***N.nubaensis* Wadi Al Ghallah SD 10-5, southern Sudan **B***N.nubaensis* Fugnido EHKS 09-01*, western Ethiopia **C***N.albertinensis* Olobodagi UG 99-23, northwestern Uganda **D***N.ugandensis* Busesa UG 99-5 (red phenotype), southeastern Uganda **E***N.ugandensis* Busesa UG 99-5 (blue/yellow phenotype), southeastern Uganda **F***N.ugandensis* Namasagali UG 99-3* (red phenotype with submarginal band in caudal fin), south-central Uganda **G***N.derhami* Ahero KEN 19-16*, western Kenya **H***N.attenboroughi* Nata TAN 93-3, north-central Tanzania **I***N.venustus* Chato TZN 19-5*, north-central Tanzania **J***N.moameensis* Mabuki TZN 19-8*, north-central Tanzania **K***N.hoermanni* Bumburi TZHK 2018-03*, central Tanzania **L***N.torgashevi* TNT 2014-04*, south-central Tanzania **M***N.streltsovi* TSTS 10-05, south-central Tanzania **N***N.itigiensis* Itigi TAN 03-8*, central Tanzania **O***N.kardashevi* Mpanda K 2011-25*, southwestern Tanzania. The fishes on the photos have a size of 45–50 mm SL (standard length). Photographs by Béla Nagy (**A, B, G, I, J–L, O**) and Brian Watters (**C–F, H, M, N**).

*Nothobranchius* spp. are small fishes, mostly reaching 30–70 mm in standard length, with only a few species achieving 100 mm or more. They show marked sexual dimorphism and dichromatism; the typically robust and colourful males contrast with the slightly smaller and dull-coloured females (Jubb 1981; Wildekamp 2004). Representative male phenotypes of the *Nothobranchiusugandensis* Wildekamp, 1994 species group are shown in Fig. 1. The male colour pattern is species-specific and thus provides an important diagnostic character for species discrimination (e.g., Jubb 1981; Nagy 2018; Nagy et al. 2020). The genus includes *N.furzeri* Jubb, 1971, the vertebrate species with the shortest lifespan recorded in captivity (less than 12 weeks), and which has emerged as a model organism for biological and molecular studies of ageing (e.g. Cellerino et al. 2016). Another species, *N.rachovii* Ahl, 1926, exhibits the lowest recorded diploid chromosome number (2n = 16) within the genus and one of the lowest diploid chromosome numbers among all karyotyped fishes (Arai 2011). With its remarkably large chromosomes, it is a convenient model for laboratory chromosome studies of fish genotoxicity (e.g. van der Hoeven et al. 1982; Krysanov 1992; Krysanov et al. 2018).

**Figure 2.**
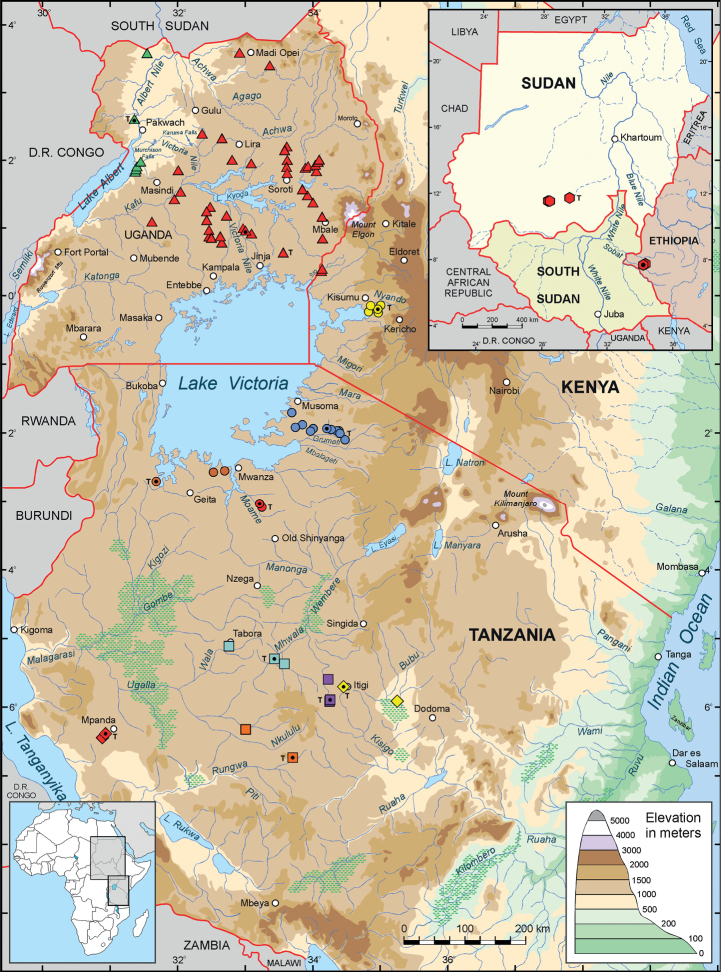
Distribution of species in eastern and northeastern Africa belonging to the *Nothobranchiusugandensis* species group: *N.albertinensis* (green triangle), *N.ugandensis* (red triangle), *N.derhami* (yellow-filled circle), *N.attenboroughi* (blue-filled circle), *N.venustus* (orange-brown-filled circle), *N.moameensis* (red-filled circle), *N.hoermanni* (blue-green square), *N.torgashevi* (purple square), *N.itigiensis* (yellow diamond), *N.streltsovi* (orange square), *N.kardashevi* (red diamond), and *N.nubaensis* (red hexagon; on inset map). T, type localities. Symbols with a black dot indicate sites of individuals used for karyotype analyses. Note that the presently known entire ranges of the respective species are shown, and individual symbols may in some cases represent multiple sites in close proximity to one another.

Phylogenetic analysis revealed that the genus *Nothobranchius* comprises a monophyletic lineage that includes seven subgenera in geographically segregated clades (van der Merwe et al. 2021). The *N.ugandensis* species group (sensu Nagy et al. 2020) belongs to the subgenus Zononothobranchius Radda, 1969. The species group currently comprises 12 members, known from the inland plateau of eastern Africa (see Table 1 and Fig. 2).

**Table 1.** Listing of all known species of the *Nothobranchiusugandensis* species group with indication of associated drainage and region of occurrence.

Species	Drainage	Region of occurrence
*N.albertinensis* Nagy, Watters et Bellstedt, 2020	Lake Albert basin and Albert Nile drainage	North-western Uganda
*N.attenboroughi* Nagy, Watters et Bellstedt, 2020	Grumeti and other small systems draining into eastern shore of Lake Victoria	Northern Tanzania
*N.moameensis* Nagy, Watters et Bellstedt, 2020	Moame and other smaller river systems draining into southern shore of Lake Victoria	
*N.derhami* Valdesalici et Amato, 2019	Nyando system northeast of Lake Victoria	South-western Kenya
*N.hoermanni* Nagy, Watters et Bellstedt, 2020	Mhwala system in the upper Wembere drainage, and the Wala system, in the Malagarasi drainage	Central Tanzania
*N.itigiensis* Nagy, Watters et Bellstedt, 2020	Upper Ruaha drainage and the Bahi Swamp	
*N.streltsovi* Valdesalici, 2016	Nkululu, tributary of the Ugalla in the Malagarasi drainage	
*N.torgashevi* Valdesalici, 2015	Wembere drainage in the endorheic Lake Eyasi basin	
*N.kardashevi* Valdesalici, 2012	Katuma system	South-western Tanzania
*N.nubaensis* Valdesalici, Bellemans, Kardashev et Golubtsov, 2009	Wadi Al Ghallah system and Khor Abu Habl system in the White Nile drainage, and the Sobat system in the Blue Nile drainage	Southern Sudan and south-western Ethiopia
*N.ugandensis* Wildekamp, 1994	Lake Victoria and Lake Kyoga basins, and Victoria Nile and Achwa drainages	Central and northern Uganda, and south-western Kenya
*N.venustus* Nagy, Watters et Bellstedt, 2020	Small stream systems as part of southwestern shore of Lake Victoria basin, and Kongwa system in the southern part of the lake	North-western Tanzania

Cytogenetic data, available for 65 *Nothobranchius* species and a taxonomically undetermined *Nothobranchius* sp. Kasenga, indicate remarkable karyotype dynamics with chromosome counts ranging from 16 to 50 (Scheel 1990; Krysanov et al. 2016; Krysanov and Demidova 2018). Sex chromosomes of the XY type have been found in two closely related species, *N.furzeri* and *N.kadleci* Reichard, 2010 (Reichwald et al. 2015; Štundlová et al. 2022), while six other representatives with scattered positions across the phylogeny possess an X_1_X_1_X_2_X_2_/X_1_X_2_Y multiple sex chromosome system (Ewulonu et al. 1985; Krysanov et al. 2016; Krysanov and Demidova 2018; Simanovsky et al. 2019). Consequently, the genus *Nothobranchius* represents an excellent model for studying processes that shape karyotype differentiation and their relevance to species diversification and reproductive isolation.

In the present study, we examined the karyotype differentiation of seven members of the *N.ugandensis* species group by conventional karyotyping. The karyotypes of the remaining five species of this group have been previously reported (Krysanov and Demidova 2018). Aiming to interpret all known cytogenetic patterns in the phylogenetic context, we constructed a phylogenetic tree based on two mitochondrial genes.

## ﻿Materials and methods

In total, we analysed thirty-three individuals belonging to seven species from the *N.ugandensis* species group (details provided in Table 2). The experiments were carried out in accordance with the rules of the Severtsov Institute of Ecology and Evolution (IEE) and approved by IEE’s Ethics Committee (orders No. 27 of November 9, 2018 and No. 55 of December 12, 2021).

**Table 2.** Number of individuals karyotyped (N), population codes and geographic coordinates for studied members of the *Nothobranchiusugandensis* species group.

Species	N	Population code	GPS coordinates
*N.albertinensis*	2 larvae	Packwach UGN 17-16	02°36.31'N, 31°23.07'E
*N.attenboroughi*	4 larvae	Mugeta TAN 17-13	01°56.77'S, 34°14.25'E
*N.derhami*	2♀/2♂	Ahero KEN 19-16	00°12.85'S, 34°57.44'E
*N.hoermanni*	4♀/2♂	Bumburi TZHK 2018-03	05°18.23'S, 33°26.07'E
*N.itigiensis*	2♀/4♂	Itigi TAN 03-8	05°41.93'S, 34°28.80'E
*N.kardashevi* *	2♀/2♂	Mpanda K 2011-25	06°22.06'S, 30°56.16'E
*N.moameensis*	2♀/2♂	Mabuki TZN 19-8	03°01.46'S, 33°12.25'E
*N.nubaensis* *	2♀/2♂	Fugnido EHKS 09-01	07°44.48'N, 34°15.03'E
*N.streltsovi* *	2♀/2♂	TNT 2014-07	06°40.87'S, 33°41.00'E
*N.torgashevi* *	3♀/4♂	TNT 2014-04	05°53.09'S, 34°17.12'E
*N.ugandensis* *	2♀/3♂	Namasagali UG 99-3	00°57.41'N, 33°01.67'E
*N.venustus*	3♀/4♂	Chato TZN 19-5	02°42.59'S, 31°43.69'E

* Data from Krysanov and Demidova (2018).

### ﻿Cytogenetic analysis

Chromosome preparations from adult individuals were obtained following Kligerman and Bloom (1977), with modifications described in Krysanov and Demidova (2018). For larvae a modified technique was used. The 1–2-week-old larvae were held in a 0.1% colchicine solution in aquarium water for 3–5 hours, then they were euthanized with an overdose of tricaine methanesulfonate (MS-222) and dissected under a Stemi 2000-C stereomicroscope (Carl Zeiss, Germany). All abdominal organs were taken for chromosome preparations. The organs were incubated with a 0.075M KCl hypotonic solution for 20 minutes and fixed in three changes of a 3:1 methanol: acetic acid solution for 20 minutes each. Finally, the fixed organs were incubated in 50–100 µL of 50% glacial acetic acid, suspended, and dropped onto hot slides (45 °C).

The chromosome spreads were air-dried, stained with 4% Giemsa solution in a phosphate buffer solution (pH 6.8) for 8 minutes and then analysed using an Axioplan 2 imaging microscope (Carl Zeiss, Germany) equipped with a CV-M4^+^CL camera (JAI, Japan) and Ikaros software (MetaSystems, Germany). At least 10 complete metaphases per individual were analysed. Final images were processed using Photoshop software (Adobe, USA). Karyotypes were arranged according to the centromere position following the nomenclature of Levan et al. (1964), but modified as metacentric (m), submetacentric (sm) and subtelocentric/acrocentric (st/a). Chromosome pairs were arranged according to their size in each chromosome category. To determine the chromosomal arm number per karyotype (nombre fondamental, NF), metacentrics and submetacentrics were considered as biarmed, and subtelocentrics/acrocentrics as monoarmed.

### ﻿Phylogenetic analyses

We constructed the phylogenetic tree for the purpose of cytogenetic data interpretation. The sequences used for the phylogenetic analysis were from Nagy et al. (2020). However, only one representative per species was chosen for this study. The phylogenetic hypothesis was based on the analysis of two mitochondrial genes *Cytochrome oxidase subunit I (COI)* and *NADH dehydrogenase 2 (ND2)*. Multiple sequence alignment was performed with Clustal Omega (Sievers et al. 2011), and the alignments of the two genes were concatenated into a single dataset of 2511 bp in length. *Nothobranchiustaeniopygus* Hilgendorf, 1891 and *N.rubroreticulatus* Blache et Miton, 1960 were selected as outgroup as representatives of closely related species groups. Phylogenetic analysis of the dataset was performed using Bayesian inference in MrBayes 3.2.7 (Ronquist and Huelsenbeck 2003). The analysis was set to Markov chain Monte Carlo simulation (mcmc) with default heating conditions. The evolutionary model for the GTR substitution model was set with gamma-distributed rate variation across sites and a proportion of invariable sites (GTR + I + I’), searching the tree space for 2 million generations starting with random trees and a sampling frequency of each 500 generations. The tree file was imported into Figtree 1.4.4. (Rambaut 2009) for tree drawing.

## ﻿Results and discussion

**Figure 3.**
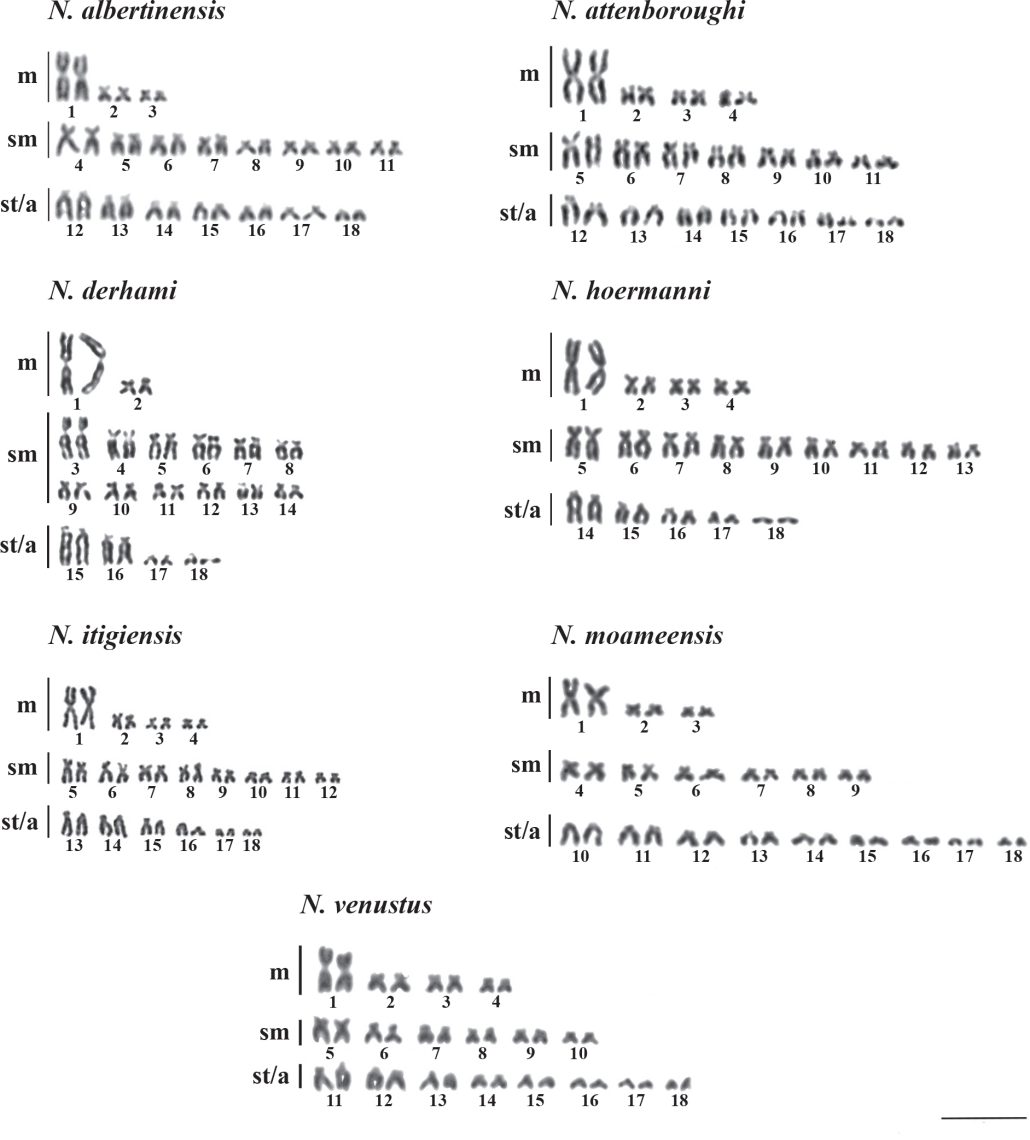
Karyotypes of seven studied members of the *Nothobranchiusugandensis* species group. Scale bar: 10 μm.

**Figure 4.**
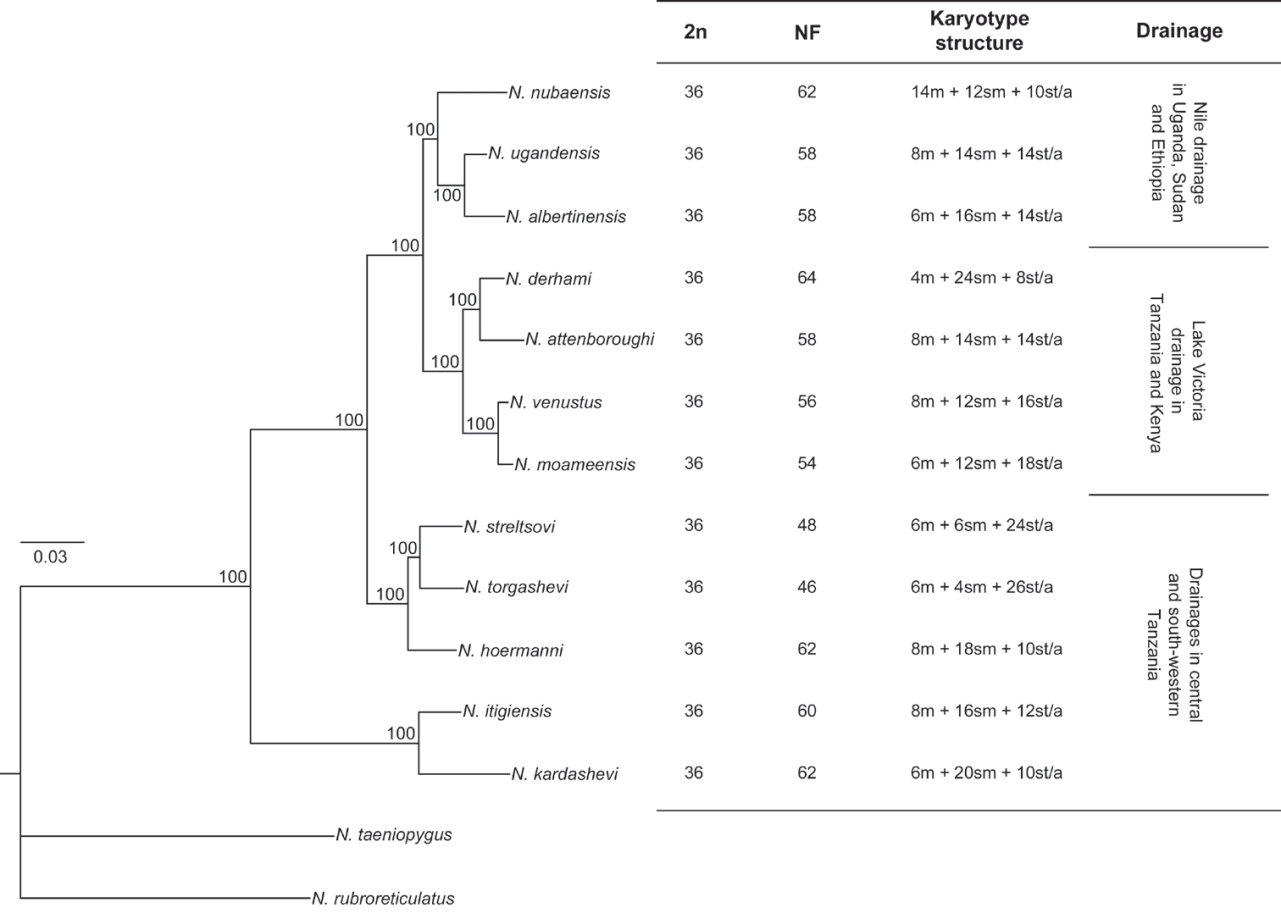
Karyotype characteristics and phylogenetic relationships, as well as associated drainage system information, for members of the *Nothobranchiusugandensis* species group. Karyotype characteristics are plotted onto the phylogenetic tree which is based on analysis of the mitochondrial molecular markers *Cytochrome oxidase subunit I (COI)* and *NADH dehydrogenase 2 (ND2)*, using Bayesian inference.

Cytogenetic characteristics (2n, NF and karyotype structure) of the analysed representatives of the *N.ugandensis* species group are shown in Fig. 3 and Table 3. Known cytogenetic data for the *N.ugandensis* species group (Krysanov and Demidova 2018 and this study) are arranged in the context of phylogenetic tree analysis in Fig. 4. All twelve species share the same 2n = 36 and the largest pair of metacentric chromosomes (pairs No. 1; Fig. 3). At the same time, the species varied considerably regarding the ratio of monoarmed (subtelocentric, acrocentric) vs. biarmed (metacentric, submetacentric) chromosomes. Accordingly, NF ranged from 54 to 64 within our seven analysed species and from 46 to 64 when considering also the species studied by Krysanov and Demidova (2018). Within our sampling, we recorded the lowest number of biarmed chromosomes (18) in *N.moameensis*, while *N.derhami* had the highest number of such chromosomes (28). All species exhibited different karyotype structures except for *N.attenboroughi* and *N.ugandensis*. Notably, these two species are widely separated geographically and belong to different clades in the molecular phylogeny (Figs 2, 4). Lastly, we did not observe consistently any type of chromosome polymorphism within our sampling and thus we also did not detect any heteromorphic sex chromosomes or the presence of multiple sex chromosome systems.

**Table 3.** Diploid chromosome numbers (2n), numbers of chromosome arms (NF) and karyotype structure of all members of *Nothobranchiusugandensis* species group.

Species	2n	NF	Karyotype structure	References
*N.albertinensis*	36	58	6m + 16sm + 14st/a	This study
*N.attenboroughi*	36	58	8m + 14sm + 14st/a	This study
*N.derhami*	36	64	4m + 24sm + 8st/a	This study
*N.hoermanni*	36	62	8m + 18sm + 10st/a	This study
*N.itigiensis*	36	60	8m + 16sm + 12st/a	This study
*N.kardashevi*	36	62	6m + 20sm + 10st/a	Krysanov and Demidova 2018
*N.moameensis*	36	54	6m + 12sm + 18st/a	This study
*N.nubaensis*	36	62	14m + 12sm + 10st/a	Krysanov and Demidova 2018
*N.streltsovi*	36	48	6m + 6sm + 24st/a	Krysanov and Demidova 2018
*N.torgashevi*	36	46	6m + 4sm + 26st/a	Krysanov and Demidova 2018
*N.ugandensis*	36	58	8m + 14sm + 14st/a	Krysanov and Demidova 2018
*N.venustus*	36	56	8m + 12sm + 16st/a	This study

According to data previously available for 66 representatives (including *N.* sp. Kasenga) (Krysanov et al. 2016; Krysanov and Demidova 2018), *Nothobranchius* killifishes display high karyotype variability. Here, we studied the karyotypes of seven members of the *N.ugandensis* species group and thus increased the number of chromosomally characterized representatives of the genus *Nothobranchius* to 73. A major finding of our survey is that all 12 studied species from the *N.ugandensis* species group maintain a stable 2n = 36 (Krysanov and Demidova 2018; this study) which contrasts with the generally extensive karyotype dynamics known for the *Nothobranchius* genus as a whole. Nevertheless, the karyotypes of the 12 species vary considerably in the proportion of monoarmed and biarmed chromosomes which is further reflected in the wide range of their NF values (46–64). Therefore, while the karyotypes of many other studied *Nothobranchius* spp. underwent frequent interchromosomal rearrangements (typically fusions and fissions) (Krysanov and Demidova 2018), karyotype differentiation in the *N.ugandensis* species group seems to be restricted to intrachromosomal structural changes that have led to shifts in the centromere positions without changes of 2n. The most probable responsible mechanisms might be pericentric inversions (i.e., two-break rearrangements where the segment between the two breaks, which is then inverted by 180° and re-inserted to the chromosome, contains the centromere) and possibly also centromere repositioning (i.e., the replacement of the old centromere by the new one located elsewhere on the chromosome; Schubert 2018). Finally, we cannot exclude the possible contribution of other relevant rearrangements such as reciprocal and non-reciprocal translocations.

The stable 2n = 36 is also shared by all but four studied representatives belonging to the subgenus Zononothobranchius (Krysanov et al. 2016; Krysanov and Demidova 2018; van der Merwe et al. 2021) which encompasses the *N.ugandensis* species group and further the *N.brieni* species group (sensu Nagy 2018), the *N.neumanni* species group (sensu Wildekamp et al. 2014), the *N.rubroreticulatus* species group (sensu van der Merwe et al. 2021), and the *N.taeniopygus* species group (sensu Watters et al. 2019). Interestingly, all representatives of the subgenus with 2n other than 36 belong to the *N.brieni* species group. Since only the *N.ugandensis* species group has been fully cytogenetically characterised (Krysanov and Demidova 2018; this study), we cannot make any general conclusions about the karyotype stability/variability in the subgenus as a whole.

*Nothobranchius* genomes are known to harbour a high amount of repetitive DNA (about 60–80 %; Reichwald et al. 2009, 2015; Cui et al. 2019; Štundlová et al. 2022) that is capable of facilitating chromosome rearrangements (Redi et al. 1990; King 1993; Li et al. 2017; Brown and Freudenreich 2021) as has recently been documented for *N.furzeri* and *N.kadleci* (Štundlová et al. 2022). As the amount and distribution of repetitive DNA may vary considerably among *Nothobranchius* species (Voleníková et al. in prep.), a hypothesis worth testing experimentally would be to determine if the species from the *N.ugandensis* species group exhibit a low proportion of clustered repeats/heterochromatin blocks in their genomes, which would correspond to a limited rate of karyotype dynamics at the interchromosomal level. A striking example of positive correlation between the stable karyotypes and low amount of repeats/constitutive heterochromatin was described in haemulid fishes (Motta-Neto et al. 2019).

It is noteworthy that the *N.ugandensis* species group, forming part of the Inland Clade, diverged approximately 4 million years ago (MYA) according to van der Merwe et al. (2021). When compared to species groups in other *Nothobranchius* clades, the *N.ugandensis* species group had sufficient time for the establishment of at least some interchromosomal rearrangements which are otherwise frequent especially in the Southern and Coastal clade (Krysanov and Demidova 2018). Nevertheless, the karyotype changes in the *N.ugandensis* species group are relatively frequent given the NF range but restricted to intrachromosomal changes only. Therefore, another hypothesis worthy of future experimental testing is whether or not any constraints related to the 3D nuclear genome architecture in species belonging to this lineage are responsible for a dramatic decrease in the probability of emergence and fixation of interchromosomal rearrangements. Intriguing examples of the interplay between chromosome rearrangements and nuclear architecture have been recently reported (Vara et al. 2021; Sidiropoulos et al. 2022; Wang et al. 2022).

Chromosome inversions are known to suppress recombination in the rearranged region but only in the heterozygous constitution (Sturtevant and Beadle 1936; Stevinson et al. 2011). While inversion heterozygotes might represent a transient populational polymorphism (King 1993), they may also be maintained by balancing selection between populations with gene flow as inversion can lock together a set of alleles of adjacent genes which may confer selective advantage for local adaptation or the evolution of complex life-history traits (Hoffmann and Rieseberg 2008; Wellenreuther and Bernathez 2018). Such cases have been reported in an increasing number of teleost species (Kirubakaran et al. 2016; Arostegui et al. 2019; Pearse et al. 2019; Wilder et al. 2020; Petrou et al. 2021). Neither our sampling, nor that of Krysanov and Demidova (2018), included individuals polymorphic for cytologically detectable inversion(s); therefore, we do not suspect that inversions might have adaptive effects in our studied system. Moreover, we found no correspondence between the karyotype variation and phylogenetic relationships (Fig. 4), nor with biogeographic distribution (Fig. 2) (discussed further below). Our data suggests independent parallel processes of karyotype differentiation within the *N.ugandensis* species group where the inversions were fixed mainly by other (e.g., neutral) processes than by natural selection. Therefore, given the structuring into small, isolated populations, the most reasonable explanation for the fixation of inversions in members of the *N.ugandensis* species group might be via random genetic drift including bottlenecks and founder effects (King 1993; Hoffmann and Rieseberg 2008; Connallon et al. 2018). The latter is consistent with the ability of killifishes to disperse and colonize new sites during major floods during the rainy season (van der Merwe et al. 2021). While the possible contribution of natural selection needs to be tested, inversions could contribute to reproductive isolation between conspecific populations and closely related species by various mechanisms (King 1993; Said et al. 2018; Villoutreix et al. 2020). The reproductive isolation might be triggered also by centromere repositioning (Lu and He 2019) which is another possible mechanism that could contribute to centromeric shifts observed in our studied species.

The *N.ugandensis* species group was recovered as monophyletic in Nagy et al. (2020) and van der Merwe et al. (2021). The topology of our phylogeny presented herein, for the purpose of comparing phylogenetic relationships with karyotype differentiation (Fig. 4), is congruent with previous results in the above-mentioned analyses. Within this group, well-defined clades, comprising the following species assemblages, exhibit strong branch support: *N.nubaensis* from the northern part of the distribution of the species group in Sudan and Ethiopia with *N.albertinensis* and *N.ugandensis* from Uganda, from the upper Nile drainage; *N.attenboroughi*, *N.derhami*, *N.moameensis* and *N.venustus* from systems associated with the near-shore zones of the Lake Victoria basin in south-western Kenya and north-western Tanzania; *N.hoermanni*, *N.torgashevi* and *N.streltsovi* from central Tanzania; and *N.itigiensis* and *N.kardashevi* from central and south-western Tanzania (Nagy et al. 2020; this study).

The biogeographic relationships among members of the *N.ugandensis* species group in central Tanzania can be explained by Palaeo-Lake Manonga, when rifting at the end of the Miocene led to ponding of the east-west rivers in northern Tanzania, forming the shallow lake basin (Nagy et al. 2020). Palaeo-Lake Manonga would have provided a connection with the Malagarasi system in western Tanzania and the currently endorheic lesser systems in central Tanzania (Harrison and Mbago 1997; Van Damme and Pickford 2003). Further, members of three species groups are distributed along a south-north axis from southern and central Tanzania to the Lake Victoria drainage in northern Tanzania. Within the *N.ugandensis* species group, the presence of *N.ugandensis* in Uganda, with *N.nubaensis* in a basal phylogenetic position in that clade, suggests an ancestral dispersal northward through Uganda and further along the Nile drainage, as the latter species is currently known from southern Sudan and south-western Ethiopia. Other species groups on the inland plateau of eastern Africa show striking similarities in distribution patterns and phylogenetic relationships, namely around an ancient Lake Manonga basin, along an east-west axis in central Tanzania, as well as northwards dispersal.

In previous studies of *Nothobranchius* (Nagy et al. 2016, 2017, 2020; Watters et al. 2019, 2020; van der Merwe et al. 2021) it was proposed that geomorphological changes separated drainages and thereby isolated populations that then speciated in peripatry and allopatry, evolving into distinct species, resulting in numerous local endemics. Rapid generation turnover in relatively small populations of these strictly seasonal fishes would have accelerated the effect of genetic drift, while during the episodes of aridity the collapse of populations may have led to population bottlenecks (Nagy et al. 2020).
